# Discovery and Investigation of Mutase-like Activity in a Phenylalanine Ammonia Lyase from *Anabaena variabilis*

**DOI:** 10.1007/s11244-018-0898-1

**Published:** 2018-01-25

**Authors:** Nicholas J. Weise, Fabio Parmeggiani, Syed T. Ahmed, Nicholas J. Turner

**Affiliations:** School of Chemistry, Manchester Institute of Biotechnology, University of Manchester, 131 Princess Street, Manchester, M1 7DN UK

**Keywords:** Biocatalysis, Ammonia lyases, β-Amino acids, Aminomutases, Enzyme selectivity

## Abstract

The effect of extended reaction times on the regio- and enantioselectivity of the phenylalanine ammonia lyase (PAL)-catalysed amination of a subset of cinnamate derivatives was investigated. This was done using a PAL from the cyanobacterium *Anabaena variabilis* and incubation in a concentrated ammonia buffer. Whilst early time point analyses revealed excellent selectivities to give mostly the well-documented (*S*)-α-amino acid products, subsequent accumulation of other regio-/stereo- isomers was seen. For many *para*-substituted substrates, the β-regioisomer, a previously-unreported product with this enzyme class, was found to become more abundant than the α-, after sufficient incubation, with slight preference for the (*R*)-enantiomer. Although attempts to tune the selectivity of the PAL toward any of the three side products were largely unsuccessful, the results provide insight into the evolutionary history of this class of enzymes and reinforce the prominence of the toolbox of specific and selective cinnamate-aminating enzymes.

**Electronic supplementary material:**

The online version of this article (10.1007/s11244-018-0898-1) contains supplementary material, which is available to authorized users.

## Introduction

**Scheme 1:**
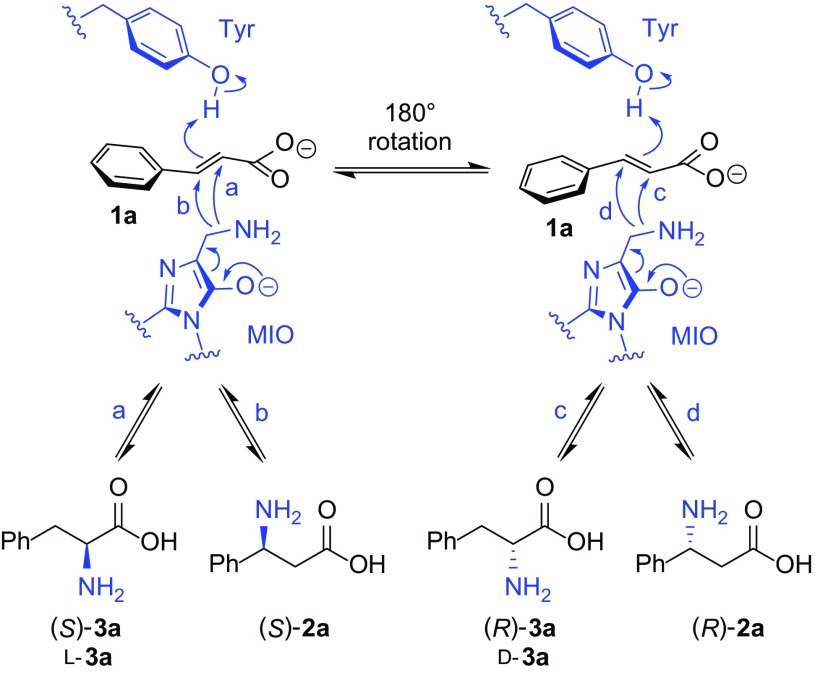
Potential reactions with MIO-dependent enzymes possessing PAL and/or PAM activity. PALs allow the reaction from (*S*)-**3a** to **1a** with the release of ammonia from the MIO, whereas PAMs are reported to catalyse the interconversion of (*S*)-**3a** and either (*R*)- or (*S*)-**2a** depending on the orientation of intermediate **1a** in the active site

In nature, enzymes with phenylalanine ammonia lyase (PAL) activity selectively bind phenylalanine (*S*)-**3a** and catalyse its deamination, forming cinnamate **1a** and ammonia [1, 2]. Relatives of these proteins have also been shown to direct subsequent reamination at the adjacent β-carbon, thus catalysing an overall isomerisation or phenylalanine aminomutase (PAM) reaction [3, 4]. The chemistry of these transformations is achieved via a family-specific active site electrophilic moiety, 4-methylideneimidazole-5-one (MIO), which allows formation of an adduct with either the primary amine of the phenylalanine substrate or ammonia to direct deamination or reamination as required (Scheme 1) [5]. The MIO acts in a concerted fashion with a conserved tyrosine residue which functions as a proton acceptor or donor and is located on an active site loop lid [6]. It has been shown through thermal activity studies of (*S*)-selective PAMs [7] and mutagenesis with an (*R*)-β-forming aminomutase [8] that the dynamics and flexibility of this inner active site loop dictate whether an overall lyase or mutase reaction occurs. This is mediated in part by positioning of the essential tyrosine in an optimal position for enzyme catalysis, with a stable, long-lived closed conformation increasing the likelihood of reamination occurring before loop movements mediating release of product(s) from the active site [7–9].

**Fig. 1:**
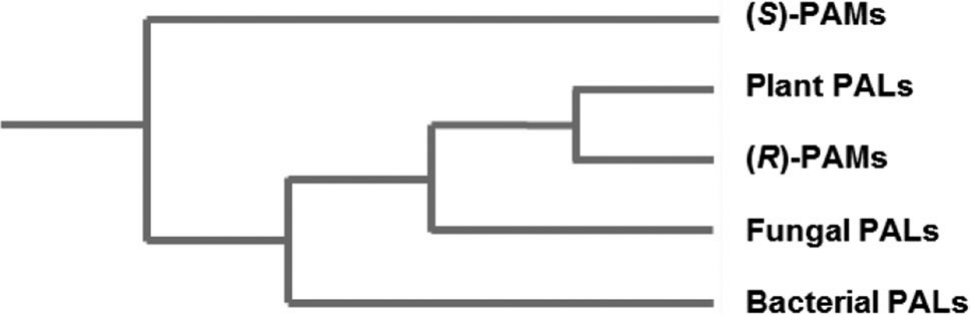
Cladogram showing the inferred evolutionary relationships between clustered groups of PAL and PAM enzyme sequences [10]

Phylogenetic analyses of various class I lyase-like sequences by Emiliani et al. [10] have previously revealed the likely evolutionary history of phenylalanine-specific enzymes within the family. Whilst enzymes with (*S*)-PAM activity are the most distant group, (*R*)-PAMs cluster within the clade of eukaryotic PALs, with which they share more sequence identity than other enzymes with PAL activity in eubacteria (Fig. 1). This indicates a probable neofunctionalisation of an ancestral PAL enzyme to allow acquisition of mutase activity in plants, with the enantiocomplementary bacterial mutases having evolved independently, far earlier and possibly from a non-PAL ancestor [10]. These data also align with reports that the active sites of PALs and (*R*)-PAMs are nearly identical, [8] whereas (*S*)-PAMs have different substrate positioning residues and are known to accommodate acrylic acids in a different binding orientation to their relatives [11]. In fact it has been theorised that this difference in binding mode of the cinnamate is the reason for the enantiocomplementarity of the two classes of aminomutase. MIO-adduct structures of an (*S*)-PAM reveal the possibility of amination/deamination on the *si* face of both carbons with little movement of the substrate [4] whereas (*R*)-selective enzymes are only thought to allow this at the α-position and instead promote rotation of a portion of the bound ligand (most probably around the C_1_–C_α_ bond), to facilitate exclusively (*R*)-β-addition [4, 12].

PALs and PAMs have both been investigated for the biocatalytic production of unnatural amino acids through the use of high pH and concentrated ammonia buffer to direct amination of various arylacrylic acids [13]. The main difference between the two enzyme classes in this respect is that enzymes with only PAL activity show production of α-phenylalanine derivatives, [14, 15] whereas characterised PAMs allow formation of both regioisomers as a mixture in most cases [16–18]. These findings imply that, in the synthetic direction, PAL enzymes accommodate cinnamate derivatives only in the α-productive binding mode, whereas (*R*)-PAMs (e.g., TwPAM from *Taxus wallichiana*) are able to sample both predicted conformers more equally. Biocatalysts featuring the (*S*)-PAM active site architecture (e.g., EncP from *Streptomyces maritimus*) seem to be able to mediate addition at either position through their single, altered trajectory of binding. However, there are examples of significant formation of the (*R*)-enantiomer of certain α-arylalanines with absolute requirement for the catalytic loop tyrosine residue. This observation has been suggested to be due to low level sampling of an (*R*)-PAM-like rotated binding mode, allowing protonation to accompany amination on the appropriate face of the substrate [19, 20]. There have also been kinetic studies with a eukaryotic PAL from *Rhodotorula graminis* in which deamination of β-phenylalanine is reported, albeit at a rate 800 times lower than with the natural α-lyase reaction [7].

Despite the striking similarities between the active sites of some PAL and PAM enzymes and the evidence of minor side activities in different ammonia lyases, the extent of this relationship with regards to selectivity has not been investigated. Herein we report the discovery of significant levels of β-amino acid formation with certain substrates using an extensively studied PAL biocatalyst with no previously identified aminomutase activity.

## Results and Discussion

A well-characterised biocatalyst, the ammonia lyase from the cyanobacterium *Anabaena variabilis* (AvPAL), was chosen for investigation, as it had already shown broad substrate scope but also imperfect enantiocontrol in some cases [19–21]. As an expansion to previous work, the enzyme was tested with a range of substrates (a panel of 21 cinnamic acid derivatives) under reaction conditions similar to those reported for the investigation of amination selectivity with a related enzyme [18] with 20 mg mL^−1^ whole cell biocatalyst at 30 °C (Table 1). The catalyst gave variable conversion with all of the substrates tested, from 2% with 4-methoxycinnamate **1u** to > 99% with 2,3,4,5,6-pentafluorocinnamate **1f**. The pattern of conversions for substrate isomers with the same ring substituent tended to follow the trend *ortho*- > *meta*- > *para*-, with the exception of the nitro- and methyl-compounds, which all gave similar values. The particularly poor acceptance of 4-methoxycinnamate **1u**, giving just 2% conversion to *O*-methyltyrosine **3u**, fits with existing observations that PAL-enzymes do not accept tyrosine well as a deamination substrate [1].

**Table 1:** AvPAL-catalysed amination of a panel of ring-substituted cinnamates **1a-u**


Subs	R	Conv. **1** (%)^a^	ee **3** (%)^b^	Ratio **2**:**3** (−)^a^
**1a**	H	64	> 99	0
**1b**	2-F	90	96	0
**1c**	3-F	78	96	0
**1d**	4-F	72	> 99	4:96
**1e**	3,5-F_2_	86	78	0
**1f**	2,3,4,5,6-F_5_	> 99	–^c^	0
**1g**	2-Cl	85	> 99	0
**1h**	3-Cl	68	> 99	0
**1i**	4-Cl	54	> 99	9:91
**1j**	2-Br	66	> 99	0
**1k**	3-Br	55	> 99	2:98
**1l**	4-Br	42	> 99	9:91
**1m**	2-NO_2_	88	52	0
**1n**	3-NO_2_	89	83	0
**1o**	4-NO_2_	83 (< 1)^d^	49	0
**1p**	2-Me	64	90	0
**1q**	3-Me	58	94	1:99
**1r**	4-Me	59	> 99	18:82
**1s**	2-MeO	58	> 99	0
**1t**	3-MeO	25	> 99	1:99
**1u**	4-MeO	2	> 99	0

^a^Determined by HPLC on a non-chiral phase

^b^Determined by HPLC on a chiral phase

^c^Not determined

^d^Conversion in control reaction without biocatalyst

The enantiomeric excess values for the (*S*)-α-amino acid products were found to be excellent (> 99%) for only 12 of the substrates tested, with significant amounts of the (*R*)-enantiomer detected for all others. The four compounds giving products with the lowest enantiopurities in this work (3,5-difluorocinnamate **1e** and nitrocinnamates **1m–o**) showed consistency with previous work on the enantioselectivity of this enzyme [19, 20]. Additional to these were the *ortho-* and *meta*-isomers with fluoro and methyl substituents (**1b, c, p** and **q**), which gave a small amount of the (*R*)-α-amino acid product. Concerning the methylcinnamates, this is the first time that substrates with an electron-rich aromatic ring have been shown to allow formation of the (*R*)-enantiomer. It is possible that this is due to different positioning of these substrates in the active site, although this does not seem to be the case with most other 2- and 3-substituted arylacrylic acids tested. The findings for the fluorinated substrates also differ from literature results, where the enantioselectivity of the equivalent reactions have been shown to be perfect. This may not have been observed before due the differences in reaction conditions between this work and other similar studies [20].

Upon HPLC analysis of the AvPAL biotransformations with the substrate panel above, it was found that 8 of the compounds gave two product peaks. By retention time comparisons with authentic standards, the additional product was confirmed to be the corresponding β-amino acid in all cases (Table 1). Reactions with the 2- and 3-bromocinnamates (**1j** and **k**) gave β:α product ratios of 2:98, while only traces of the β-product were detectable with 3-methyl- and 3-methoxy- compounds (**1q** and **t**). For the 4-halo- and 4-methyl- substrates (**1d, i, l** and **r**) more β-formation was observed (between 4:96 and 18:82). The occurrence of β-addition with AvPAL seemed to be specific to these compounds, with no evident relationship to substrate conversion. For instance, the highest converted of the β-forming substrates **1d** gave a lower ratio than **1r** (with the least selective ratio of 18:82), but a higher ratio than the lowest converted substrate **1t**.

Evidence of varied regioselectivity in amination reactions with AvPAL is surprising as this enzyme is an ammonia lyase and has never been shown to have discernible aminomutase activity [1]. Ammonia addition at both positions has only been observed in the enzyme family before with EncP from *Streptomyces maritimus* and TwPAM from *Taxus wallichiana*, enzymes known to catalyse aromatic amino acid isomerisation reactions in the absence of ammonia. These wild-type aminomutases have also both been shown to give roughly equal mixtures of phenylalanine regioisomers in amination reactions with cinnamate, [16, 18] whereas PALs are reported to give only α-phenylalanine [14, 22]. However, the previously mentioned deamination of β-phenylalanine by RgPAL [7] highlights the fact that the reverse reaction (i.e., amination to form β-amino acids) may be possible using PAL enzymes, even with the cinnamate as starting material. Interestingly, the subset of compounds where β-amino acids were detected as side products with AvPAL was also found to give greater β:α ratios than the unsubstituted natural acrylic acid (**1a**) with EncP in previous investigations. However EncP also gave similar regioselectivity changes with 2- and 4-methoxycinnamate (**1s** and **u**) not observed here [18]. The latter substrate presumably gives no detectable quantities of β-product in this instance due to it being poorly accepted by AvPAL. Oddly, **1s** shows no detectable side product despite a moderate conversion of 58%. Comparison of the percentage of β-formation with substituent- and position- specific core electron binding energy shift (ΔCEBE) values [23] for each substrate showed correlation for only a limited number of substrates (see ESM). This implies that substrate positioning in AvPAL mostly counteracts any electronic effects from the substrates. This is in contrast to similar analyses with EncP, where electronic effects were found to influence regioisomer production [18].

**Fig. 2:**
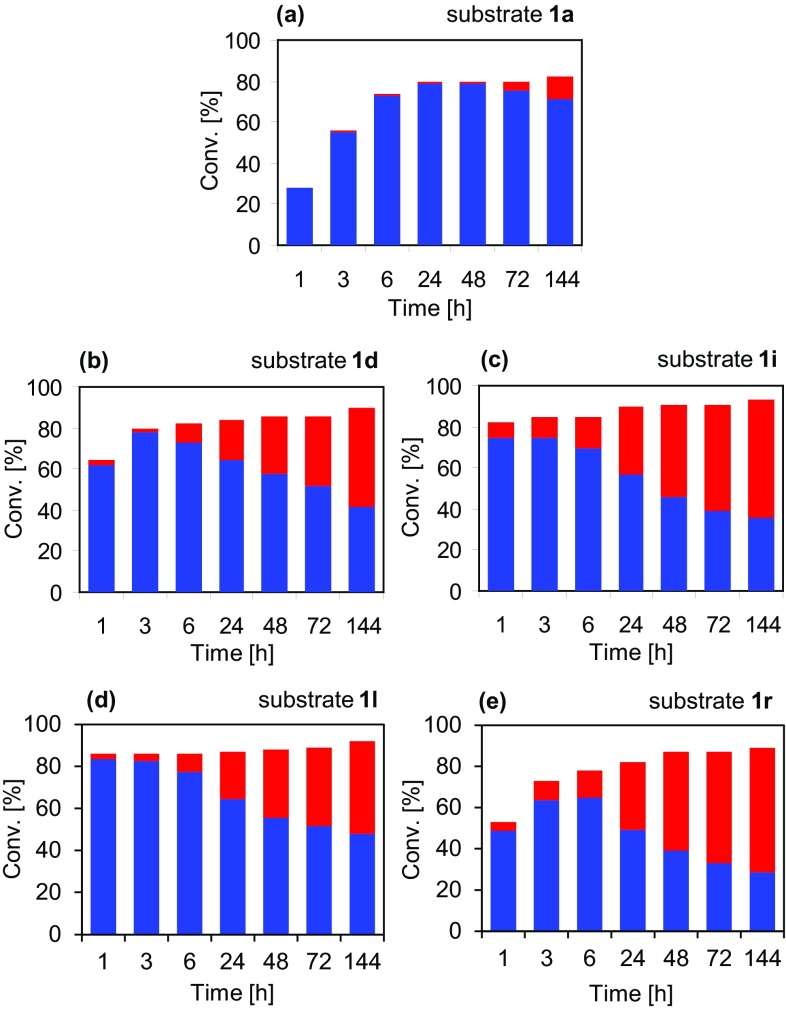
Time course experiments of the AvPAL-catalysed synthesis of β- and α-amino acids from substrates (**1a, d, i, l** and **r**). Blue bars represent the proportion of α-product **3**, red bars the proportion of β-product **2**

To test the relative rates of regioisomer production for the reaction, the biotransformations of the four substrates giving the highest β:α product ratios (**1d, i, l** and **r**), along with **1a** as a control experiment, were repeated. For these reactions, the catalyst loading was increased to 50 mg mL^−1^ and time points taken over 6 days (Fig. 2). The biocatalyst was found to give maximal conversion of all five substrates (80–90%) within the first 24 h of the reaction. Although conversion remained more or less constant after this, the levels of each amino acid fluctuated significantly, with initial production of mostly the α-regioisomer followed by gradual accumulation of the β-product. For three of the substrates (**1d, i** and **l**) the level of the β-amino acid was found to overtake that of the classical PAL product, along with the lower final β:α ratio (48:52) for **1r** and even the control substrate **1a** showing significant side-product after 48 h. This production of one regioisomer first followed by the other is very different to time course experimental results with TwPAM, where the β:α ratio has been shown to remain more or less constant, even with increasing conversion over time [8]. This is most likely due to the difference in regioselectivity between PAL and PAM enzymes, whereby different kinetic constraints are imposed on the β- and α-addition reactions in combination with an apparent preference for the β-regioisomer under the reaction conditions used in this work. The production of predominantly β-amino acid by the end of the time course experiment may be due to this regioisomer being in some way favoured thermodynamically, possibly by higher solubility in the ammonium sulphate buffer or by some product stabilising interaction with one or more components of the reaction mixture. It is also conceivable that the rate of deamination of the β-product is so slow compared to its amination (and compared to the cognate rates for the α-regioisomer) that it becomes trapped and builds up over time as more and more β-promoting binding modes are sampled.

Furthermore, in our recent effort to prepare a broad panel of arylalanines from arylaldehydes, [24] exploiting the AvPAL-mediated hydroamination as a key step, the same behaviour was observed. For several of the pyridylacrylic acids tested, albeit under different conditions than those reported in this study, an additional amino acid product was apparent from the NMR analysis after prolonged incubation with the biocatalyst, reaching in some cases a consistent proportion of the crude mixture (see ESM). Control over reaction parameters such as incubation time and whole cell loading allowed us to suppress completely the formation of the side-product (see ESM), eliminating the need to separate it after reaction work-up. However, upon further investigation, this side-product has been identified as the β-amino acid, adding further evidence to the findings reported here.

To test the enantioselectivity of the AvPAL amination reaction at both positions, the five biotransformations were run again for 4 days and the results analysed by HPLC on both chiral and non-chiral stationary phases (Table 2a). Conversions and product ratios were found to be comparable to data from earlier experiments. Interestingly, after the longer reaction time there were detectable amounts of (*R*)-α-amino acid with all five substrates, giving ee values of 80–92% (*S*). This, combined with some lower ee values with methyl- and fluoro- substrates in the initial amination experiments, implies that the formation of the ‘unnatural’ (*R*)-enantiomer may be possible with all substrates, if the reaction proceeds for long enough. The (*R*)-selective amination at the α-position seemed to be faster with substrates with an electron-deficient ring, but did not occur exclusively with them. This may explain why these findings have only been reported with such compounds under the shorter reaction times (and with higher substrate concentrations) in preceding work [19, 20].

**Table 2:** AvPAL-catalysed amination of cinnamates **1a, d, i, l** and **r** for chiral analysis (a) and AvPAL-catalysed deamination of the corresponding enantiopure α-amino acids (*S*)-**3a, d, i, l** and **r** (b)


(a) Subs	R	Conv. **1** (%)^a^	Ratio **2**:**3** (−)^a^	ee (*R*)-**2** (%)^b^	ee (*S*)-**3** (%)^b^
**1a**	H	73	25:75	14	92
**1d**	4-F	83	66:34	16	91
**1i**	4-Cl	84	69:31	8	85
**1l**	4-Br	81	67:33	–^c^	–^c^
**1r**	4-Me	83 (< 1)^d^	72:28	44	80


(b) Subs	R	Conv. (%)^a^
(*S*)-**3a**	H	> 99
(*S*)-**3d**	4-F	> 99
(*S*)-**3i**	4-Cl	> 99
(*S*)-**3l**	4-Br	> 99
(*S*)-**3r**	4-Me	> 99

^a^Determined by HPLC on a non-chiral phase

^b^Determined by HPLC on a chiral phase

^c^Not determined

^d^Conversion in control reaction without biocatalyst

Enantiomeric excess values for four of the five β-products (**2a, d, i** and **r**) could be obtained with the HPLC method used. These were found to be poor but each showed an excess of the (*R*)-configured amino acid with values ranging from almost racemic (8% ee for **2i**) to moderately enriched (44% ee for **2r**). This suggests that the ammonia lyase active site imparts selectivity at both positions in a mode similar to the (*R*)-selective aminomutase TwPAM, possibly through the same distinct binding modes for (*S*)-α- and (*R*)-β-addition. Comparison of the excellent enantioselectivity and more constant β:α-ratio of TwPAM with the same parameters for AvPAL adds further evidence to the hypothesis that the (*R*)-β-productive binding mode in the second enzyme is less favoured. To confirm this catalytic similarity, five further biotransformations were set up using the same reaction conditions as before but with 1 mM of the (*S*)-α-amino acids (**3a, d, i, l** and **r**) instead of the counterpart acrylic acids, and 0.1 M borate buffer (pH 8.3) to discourage reamination (Table 2b). Unsurprisingly, AvPAL was found to deaminate all five amino acids fully after a 22 h incubation. The ability of an ammonia lyase to allow both deamination of (*S*)-α-phenylalanine and derivatives, and β-amination with slight (*R*)-selectivity, albeit under different conditions, points to the evolutionary origins of (*R*)-selective PAM activity. Such artefacts had presumably not been evident before due to the flexible AvPAL inner loop preventing retention of lyase products in the active site for long enough to reveal true mutase activity in studies with amino acid starting material.

The occurrence of enantioselective β-amino acid formation by AvPAL presented an opportunity to engineer a biocatalyst for the formation of (*R*)-β-arylalanines from cinnamate derivatives. This has been previously carried out by Janssen et al. with TwPAM as a starting template. In their study, the enzyme of choice already had excellent enantioselectivity but formed a regioisomeric mixture of (*R*)-β- and (*S*)-α-phenylalanine derivatives with most substrates tested and suffered very low turnover rates for all. These hurdles were overcome by directed evolution of the active site and extensive rational design of the inner active site loop. Saturation libraries across three pairs of active site residues (approximately 1200 variants) yielded a handful of hits with more desirable β-selectivity, the best of which being TwPAM-Q319M [25]. After initial computational modelling of the complex dynamics of loop opening in TwPAM, mutations were introduced to remove any molecular constraints on this process, of which one, TwPAM-R92S, was effective [8]. In a subsequent study, additional dynamics simulations combined with loop hydrophobicity studies and comparison to a related ammonia lyase were used to create more variants with increased amination rates [9].

Due to the complexity of studying protein dynamics with a view to enzyme redesign, it is desirable to have a starting point where inner active site loop properties are already suited to amination reactions. As AvPAL already had good turnover rates, courtesy of its lyase-like catalytic cycle at ambient temperature, work was initiated on the enhancement of the potentially useful side activities discovered in this enzyme. To begin with, two amino acid substitutions were selected for introduction into the enzyme. The first was F107C, a speculated selectivity switch between mutases and lyases, [8] chosen to make the active site of AvPAL more similar to that of (*R*)-selective aminomutases. It was hoped that this change would improve the enantio- and initial regioselectivity of the enzyme to make it more like the wild-type TwPAM. The second variant to be made was AvPAL-R317K, a substitution homologous to that made in regioselectivity studies with the (*S*)-selective aminomutase EncP [18]. This variant had been shown to have increased β-selectivity and so it was predicted that the same mutation in AvPAL might have a comparable effect.

**Fig. 3:**
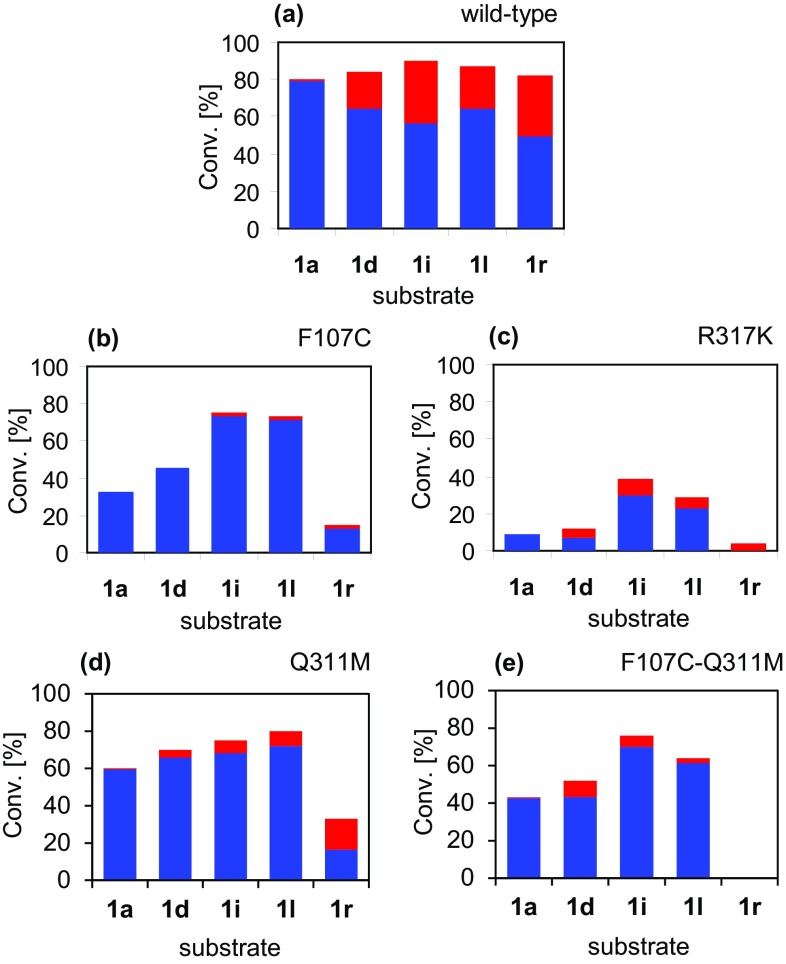
Amination of cinnamates **1a, d, i, l** and **r** catalysed by wild-type AvPAL and active site variants F107C, R317K, Q311M and F107C-Q311M. Blue bars represent the proportion of α-product **3**, red bars the proportion of β-product **2**

The variant proteins were produced for use in whole-cell biocatalytic conversions of various arylacrylic acids as before (Fig. 3a–c). In this case the classically β-forming substrates (**1l, i, d** and **r**) were tested, along with **1a** as a control substrate. Reaction conditions were kept the same as before with 50 mg mL^−1^ to ensure conversion was seen after unoptimised protein production conditions. AvPAL-F107C showed conversion with all substrates tested, but the regioselectivity was not found to be affected as predicted. Within the 22 h reaction time no β-amino acids were seen for biotransformations with **1a** and **1d**. The R317K variant showed low conversions with most substrates. Nonetheless, β-amination was observed with all four *para-*substituted substrates. The regioselectivity for the reaction of **1r** was particularly interesting as only β-amino acid could be detected as product, although with very low conversion (4%). Unfortunately, a combination of poor conversions and/or low regioselectivity meant that determination of ee values for the β-products formed in this suite of reactions was not possible. It is worth mentioning that the R317K variant was found to be difficult to handle as a whole cell biocatalyst, as freeze-thawing the cells or storing them at 4 °C for subsequent biotransformations resulted in a complete loss of enzyme activity. However, regrowing the protein production culture led to similar activity as the first cell batch and the same storage difficulties. This could be akin to the drop in temperature optimum and substrate conversions seen when comparing the EncP-R299K and wild-type EncP whole cell biocatalysts in previous studies [18].

In the interests of ascertaining possible synergistic effects of site-directed mutagenesis the AvPAL double variant AvPAL-F107C-R317K was also made and tested with the same panel of compounds as the single variants. The enzyme was found to have no activity with any of the compounds under the same reaction conditions. The presence of the protein in the whole cells was confirmed by observation of an overproduced band of correct molecular weight in an SDS–PAGE analysis following cell lysis. Inspired by the directed evolution efforts detailed with TwPAM, an additional AvPAL variant was created. AvPAL-Q311M was designed in the image of the TwPAM-Q319M variant, which had been shown to give much improved β-regioselectivity with cinnamate and a small group of derivatives [25]. It was predicted that this active site alteration might have a similar effect on the regioselectivity of AvPAL to the R317K mutation, but without the stability/activity issues incurred by transferring mutations between these more distantly related family members. Initial tests with AvPAL-Q311M were performed as for the time course experiments and with the same substrates for 22 h (Fig. 3d). However, regioselectivity was scarcely found to be better than initial substrate screening studies with the wild-type enzyme. None of the compounds gave any improvement in regioselectivity over the best β:α ratios observed in other biotransformations in this work. Again, the combination of conversion and β:α ratio for each substrate contravened determination of β ee values. The Q311M substitution was, however found to allow multiple freeze–thaw cycles of the whole cell biocatalyst whilst retaining activity. In the same vein as with the previous double variant, both Q311M and F107C were introduced into the final PAL protein via plasmid mutagenesis. This created an enzyme with an identical active site to TwPAM-Q319M. Testing of this final variant with the five substrates from before (Fig. 3e) gave moderate conversions with four of them, but again no improvements in β-selectivity (the best substrate being 4-fluorocinnamate **1d**, with 52% conversion and a 17:83 β:α product ratio). In this case neither β- nor α-amino acid product could be detected after the 22 h reaction time with the 4-methylated compound **1r**.

## Conclusions

Investigation of the phenylalanine ammonia lyase from *Anabaena variabilis* (AvPAL) in the amination of various arylacrylic acids has allowed discovery of previously undetected aminomutase-like activity in this enzyme. The gradual build-up of the newly identified, enantioenriched β-amino acid product of this enzyme is possibly indicative of kinetically disfavoured side activity, a feature which may have been present in ancestral PAL enzymes allowing the subsequent evolution (*R*)-selective phenylalanine aminomutases. The findings also demonstrate the ability of the same parent enzyme to produce all of the four possible isomers of phenylalanine derivatives from a common starting material, albeit at different relative rates. The difference in selectivity between AvPAL variants and closely related enzymes with aminomutase activity (TwPAM and variants), even those with identical substrate contacting residues, further highlights the poorly understood mechanisms of mutase–lyase selectivity. It also emphasises the importance of non-active site molecular determinants in conferring, enhancing or even hindering enzyme function and reinforces the use of various natural PAL and PAM enzymes to impart selectivity rather than divergent engineering from a single starting point.

## Electronic supplementary material

Below is the link to the electronic supplementary material.

Supplementary material 1 (DOCX 326 KB)
