# Supplementary material for: Discovery and Investigation of Mutase-like Activity in a Phenylalanine Ammonia Lyase from Anabaena variabilis
Source: Top Catal. 2018 Jan 25;61(3):288–95. doi: 10.1007/s11244-018-0898-1 (PMC6413883; doi:10.1007/s11244-018-0898-1)
Supplement: Supplementary file 1 — Supplementary material 1 (DOCX 326 KB) [file 11244_2018_898_MOESM1_ESM.docx]

**ELECTRONIC SUPPLEMENTARY MATERIAL**

**Discovery and Investigation of Mutase-like Activity
in a Phenylalanine Ammonia Lyase from *Anabaena variabilis***

Nicholas J. Weise,^1^ Fabio Parmeggiani,^1^ Syed T. Ahmed^1^ and Nicholas J. Turner*^,1^

^1^ Manchester Institute of Biotechnology, School of Chemistry, University of Manchester,
131 Princess Street, Manchester, M1 7DN, United Kingdom

Table of contents

[General methods S2](#_Toc486338222)

[Site directed mutagenesis S2](#_Toc486338223)

[Biocatalyst preparation and biotransformation procedures S3](#_Toc486338224)

[Correlation between core electron binding energy and α- and β- products S3](#_Toc486338225)

[Time-course experiments with compounds **1a**, **1d**, **1i**, **1l** and **1r** S4](#_Toc486338226)

[Deamination/amination of substrates **3a**, **3d**, **3i**, **3l** and **3r** S4](#_Toc486338227)

[Amination of *ortho*- and *para*- substrates using AvPAL variants F107C, R317K, Q311M and the F107C/Q311M double mutant S5](#_Toc486338228)

[Non-chiral HPLC analysis S6](#_Toc486338229)

[Chiral HPLC analysis S7](#_Toc486338230)

[Preparative-scale production of β-amino acids with pyridylalanine substrates S8](#_Toc486338231)

# General methods

All reagents were obtained at analytical grade and solvents at HPLC grade from Sigma-Aldrich, AlfaAesar or Fisher Scientific and used without further purification unless noted. Reference standards of optically pure amino acids were used as provided by Sigma-Aldrich, AlfaAesar, or PepTech Corp. Plasmid DNA extraction from whole cell cultures was performed with the QIAprep Spin miniprep kit (Qiagen), according to the manufacturer’s protocol. DNA sequencing was outsourced to MWG Eurofins. *E. coli* DH5α was used as a cloning host for plasmid production for all DNA manipulation and sequencing efforts, *E. coli* BL21(DE3) was as an expression host for plasmid DNA to mediate protein production. Chemically competent *E. coli* cells (both DH5α and BL21(DE3) strains) were purchased from New England Biolabs and transformations with plasmid DNA performed according to the procedures provided. Solid and liquid media were supplemented with ampicillin (100 μg mL^–1^ final concentration) and prepared by addition of agar (1.5% w/v) to liquid media were appropriate. The pET-16b-AvPAL plasmid (pET-16b vector containing the gene encoding AvPAL codon-optimised for *E. coli*) was obtained as reported previously.^1, 2^

# Site directed mutagenesis

Missense mutations were introduced in the pET-16b-AvPAL template using the Phusion site-directed mutagenesis kit (Thermo Scientific) according to the manufacturer’s methods. Polymerase chain reactions (PCR) were performed using an Eppendorf Mastercycler Gradient with the following primers (mutated bases underlined):

F107C_Fw = 5’-CTGGTTTGGTGCCTGAAAACCGG-3’;

F107C_Rv = 3’-CCGTTCGCTTGACGTCTGGTTA-5’;

R317K_Fw = 5’-TTATAGCCTGAAATGTCTGCC-3’;

R317K_Rv = 3’-GTACTTGACTAGGTCCTAGC-5’;

E311M_Fw = 5’-CTGATCATGGATCGTTATAGCC-3’;

E311M_Rv = 3’-GTACTAATAGCACTAGTACTT-5’.

The PCR conditions used in each case were as follows: 98 °C (30s), [98 °C (15 s), 58 °C (30 s), 98 °C (150 s)] x 30, 72 °C (300 s). The blunt PCR product was ligated to form a mutated plasmid with subsequent transformation into E. coli DH5α for vector production, extraction and sequencing. The samples found to contain the desired mutation(s) were transformed into E. coli BL21(DE3) to allow variant biocatalyst production.

# Biocatalyst preparation and biotransformation procedures

LB medium (5 mL, supplemented with ampicillin - final concentration 100 μg mL^-1^) was inoculated with a single colony of E. coli BL21(DE3) containing a pET-16b-AvPAL plasmid with the appropriate mutation and grown for 16h at 37 °C and 250 rpm. The starter culture was used to inoculate LB-based autoinduction medium28 (800 mL, supplemented with ampicillin - final concentration 100 μg mL^-1^) and incubated at 18 °C and 250 rpm for 4 days. The cells were harvested by centrifugation (4000 rpm, 12 min) and stored at -20 °C until further use. The whole cells biocatalyst was thawed at room temperature and resuspended in a solution of substrate (1 mM) and (NH_4_)_2_SO_4_ (4 M, pH 8.3) in a total volume of 1.0 mL. The mixture was incubated at 30°C and 250 rpm for 22 h unless otherwise stated. Biotransformation samples were mixed with an equal volume of MeOH, vortexed, and centrifuged (13000 rpm, 3 min) to separate the whole cell catalyst. The supernatant was transferred to a 0.45 μm filter vial and used directly for HPLC analysis.

# Correlation between core electron binding energy and α- and β- products

**
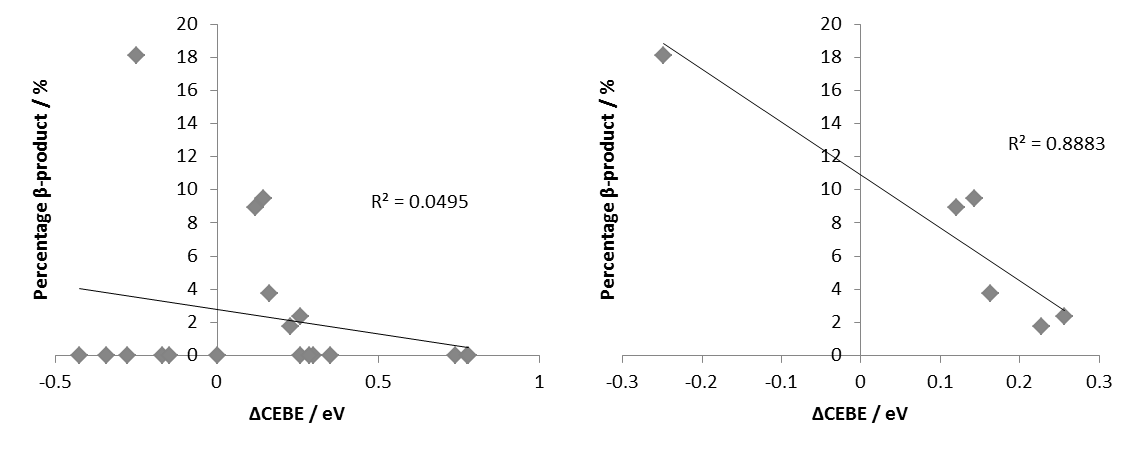
**

**Figure S1.** The dependence of β- vs. α-amination by AvPAL on the core electron binding energy shift (ΔCEBE) due to substrate ring substituents. The plot on the left includes all reaction values, whereas the plot on the right includes only reactions were β-amino acid products are observed as more than traces.

# Time-course experiments with compounds 1a, 1d, 1i, 1l and 1r

**Table S1.** Time course experiments of the AvPAL-catalysed synthesis of β- and α-amino acids from cinnamate (**1a**), 4-fluorocinnamate (**1d**), 4-chlorocinnamate (**1i**), 4-bromocinnamate (**1l**) and 4-methylcinnamate (**1r**).

| **Time / h** | **Conv. (1a)** | **2a:3a** | **Conv. (1d)** | **2d:3d** | **Conv. (1i)** | **2i:3i** | **Conv. (1l)** | **2l:3l** | **Conv. (1r)** | **2r:3r** |
| --- | --- | --- | --- | --- | --- | --- | --- | --- | --- | --- |
| **0.75** | 28% | <1:99 | 64% | 3:97 | 82% | 9:91 | 86% | 3:97 | 53% | 8:92 |
| **2.25** | 56% | <1:99 | 80% | 3:97 | 85% | 12:88 | 86% | 4:96 | 73% | 13:87 |
| **5.25** | 74% | <1:99 | 82% | 11:89 | 85% | 18:82 | 86% | 10:90 | 78% | 17:83 |
| **22** | 80% | <1:99 | 84% | 23:77 | 90% | 37:63 | 87% | 26:74 | 82% | 40:60 |
| **47** | 80% | 2:98 | 86% | 33:67 | 91% | 50:50 | 88% | 37:63 | 87% | 55:45 |
| **72** | 80% | 6:94 | 86% | 40:60 | 91% | 57:43 | 89% | 42:58 | 87% | 62:38 |
| **145** | 82% | 13:87 | 90% | 54:46 | 93% | 62:38 | 92% | 48:52 | 89% | 68:32 |

# Deamination/amination of substrates 3a, 3d, 3i, 3l and 3r

**Table S2.** AvPAL-catalysed deamination of ring-substituted (*S*)-α-amino acids **3** and amination of the corresponding cinnamate derivatives **1**.

| **R** | **Conv.^a^** | **Conv.^b^** | **2** | **:** | **3** | **ee of 2** | **ee of 3** |
| --- | --- | --- | --- | --- | --- | --- | --- |
| **H** | >99% | 73% | 25 | : | 75 | 14% (*R*) | 92% (*S*) |
| **4-F** | >99% | 83% | 66 | : | 34 | 16% (*R*) | 91% (*S*) |
| **4-Cl** | >99% | 84% | 69 | : | 31 | 8% (*R*) | 85% (*S*) |
| **4-Br** | >99% | 81% | 67 | : | 33 | n.d. | n.d. |
| **4-CH_3_** | >99% | 83% (<1%)^c^ | 72 | : | 28 | 44% (*R*) | 80% (*S*) |

^a^ Deamination of (*S*)-α-phenylalanine derivatives to the corresponding cinnamates **1**. Exp. cond. 100 mM borate buffer pH 8.3, 30°C, 22 h. ^b^AvPAL catalysed hydroamination of cinnamic acids **1** to a mixture of **2** and **3**.

^c^ Conversion in control reaction without biocatalyst. Exp. cond. 4 M (NH_4_)_2_SO_4_ pH 8.3, 30°C, 22 h.

n.d. = not determined

# Amination of *ortho*- and *para*- substrates using AvPAL variants F107C, R317K, Q311M and the F107C/Q311M double mutant

**Table S3.** Amination of cinnamate and a selection of 2- and 4-ring-substituted derivatives catalysed by two AvPAL single active site variants (F107C and R317K).

|  | | **AvPAL-F107C** | | | | **AvPAL-R317K** | | | |
| --- | --- | --- | --- | --- | --- | --- | --- | --- | --- |
| **1** | **R** | **Conv. (1)** | **2** | **:** | **3** | **Conv. (1)** | **2** | **:** | **3** |
| **1a** | **H** | 33% | <1 | : | 99 | 9% | <1 | : | 99 |
| **1j** | **2-Br** | 27% | <1 | : | 99 | 7% | <1 | : | 99 |
| **1g** | **2-Cl** | 62% | <1 | : | 99 | 12% | <1 | : | 99 |
| **1b** | **2-F** | 78% | <1 | : | 99 | 26% | 15 | : | 85 |
| **1p** | **2-CH_3_** | 6% | <1 | : | 99 | <1% | - | : | - |
| **1l** | **4-Br** | 73% | 2 | : | 98 | 29% | 22 | : | 78 |
| **1i** | **4-Cl** | 75% | 2 | : | 98 | 39% | 23 | : | 77 |
| **1d** | **4-F** | 46% | <1 | : | 99 | 12% | 39 | : | 61 |
| **1r** | **4- CH_3_** | 15% | 13 | : | 87 | 4% | >95 | : | 5 |

*.*

**Table S4.** Amination of cinnamate and a selection of 4-ring-substituted derivatives catalysed by an AvPAL single active site variant (Q311M).

| **1** | **R** | **Conv. (1)** | **2** | **:** | **3** |
| --- | --- | --- | --- | --- | --- |
| **1a** | **H** | 60% | <1 | : | 99 |
| **1d** | **4-F** | 70% | 6 | : | 94 |
| **1l** | **4-Br** | 75% | 9 | : | 91 |
| **1i** | **4-Cl** | 80% | 10 | : | 90 |
| **1r** | **4- CH_3_** | 33% | 50 | : | 50 |

**Table S5.** Amination of cinnamate and a selection of 4-ring-substituted derivatives catalysed by an AvPAL double active site variant (F107C / Q311M).

| **1** | **R** | **Conv. (1)** | **2** | **:** | **3** |
| --- | --- | --- | --- | --- | --- |
| **1a** | **H** | 43% | <1 | : | 99 |
| **1d** | **4-F** | 52% | 17 | : | 83 |
| **1l** | **4-Br** | 64% | 4 | : | 96 |
| **1i** | **4-Cl** | 76% | 8 | : | 92 |
| **1r** | **4-CH_3_** | <1% | - | : | - |

# Non-chiral HPLC analysis

Reverse phase HPLC analyses were performed on an Agilent 1200 Series system with a G1379A degasser, a G1312A binary pump, a G1329 autosampler unit, a G1316A temperature controlled column compartment and a G1315B diode array detector. An external column cooling jacket was employed instead of the temperature controlled column compartment when temperatures below 0^o^C were required. Conversion and product ratios were calculated from peak areas from analyses on a ZORBAX Extend-C18 column (50 mm × 4.6 mm × 3.5 μm Agilent), using response factors where required. Mobile phase: NH_4_OH buffer (0.35% w/v, pH 10.0) / 10-30% MeOH. Flow rate: 1 mL min^–1^. Temperature: 40°C. Detection wavelength: 210 nm. Peaks were assigned via comparison with the retention times of authentic standards. Fractions (100-200 µL) of the separated amino acid products were collected from these analytical runs for further chiral analyses.

**Table S6.** HPLC conditions and retention times for non-chiral analyses.

| **Compound** | | **MeOH [%]** | **Temp.**  **[°C]** | **Retention time**  **[min]** | | |
| --- | --- | --- | --- | --- | --- | --- |
|  | R |  |  | **2** | **3** | **1** |
| **a** | H | 10 | 40 | 1.8 | 2.3 | 5.4 |
| **b** | 2-F | 10 | 40 | 2.2 | 2.8 | 7.2 |
| **c** | 3-F | 10 | 40 | 2.4 | 3.0 | 7.7 |
| **d** | 4-F | 10 | 40 | 2.3 | 2.7 | 7.1 |
| **e** | 3,5-F_2_ | 20 | 40 | 2.1 | 2.5 | 6.4 |
| **f** | 2,3,4,5,6-F_5_ | 30 | 40 | 1.7 | 2.6 | 6.8 |
| **g** | 2-Cl | 20 | 40 | 2.5 | 3.0 | 7.4 |
| **h** | 3-Cl | 30 | 40 | 1.8 | 2.2 | 4.8 |
| **i** | 4-Cl | 30 | 40 | 1.9 | 2.3 | 5.0 |
| **j** | 2-Br | 30 | 40 | 1.8 | 2.1 | 4.3 |
| **k** | 3-Br | 30 | 40 | 2.2 | 2.8 | 6.2 |
| **l** | 4-Br | 30 | 40 | 2.3 | 2.9 | 6.5 |
| **m** | 2-NO_2_ | 10 | 40 | 2.3 | 3.9 | 5.9 |
| **n** | 3-NO_2_ | 10 | 40 | 2.2 | 3.1 | 6.8 |
| **o** | 4-NO_2_ | 10 | 40 | 1.8 | 2.8 | 6.1 |
| **p** | 2-CH_3_ | 20 | 40 | 2.1 | 2.8 | 6.6 |
| **q** | 3-CH_3_ | 20 | 40 | 2.5 | 3.2 | 7.7 |
| **r** | 4-CH_3_ | 20 | 40 | 2.4 | 3.5 | 7.1 |
| **s** | 2-OCH_3_ | 20 | 40 | 1.7 | 2.3 | 4.5 |
| **t** | 3-OCH_3_ | 20 | 40 | 1.5 | 1.9 | 4.0 |
| **u** | 4-OCH_3_ | 10 | 40 | 1.7 | 2.2 | 6.4 |
|  |  |  |  |  |  |  |

# Chiral HPLC analysis

Enantiomeric excesses were measured using a CROWNPAK CR(+) HPLC column, (150 mm x 4 mm x 5 µm, Daicel). Mobile phase: aq. HClO_4_ (1.14% w/v, pH 2.0) / MeOH. Flow rate: 1 mL min^–1^for runs at 25^o^C and 0.5 mL min^–1^ for runs at -8^o^C. Detection wavelength: 210 nm. Peaks were assigned via comparison with chromatograms available in the literature.^3^

**Table S7.** HPLC conditions and retention times for chiral analyses of **3**.

| **Compound** | | **MeOH**  **[%]** | **Temp.**  **[°C]** | **Retention time**  **[min]** | |
| --- | --- | --- | --- | --- | --- |
|  | R |  |  | **(*R*)*-*enantiomer** | **(*S*)*-*enantiomer** |
| **3a** | H | 4 | 25 | 5.0 | 6.3 |
| **3b** | 2-F | 4 | 25 | 5.8 | 7.5 |
| **3c** | 3-F | 4 | 25 | 6.5 | 8.3 |
| **3d** | 4-F | 4 | 25 | 6.6 | 8.1 |
| **3e** | 3,5-F_2_ | 4 | 25 | 7.1 | 8.9 |
| **3f** | 2,3,4,5,6-F_5_ | 4 | 25 | – | – |
| **3g** | 2-Cl | 14 | 25 | 8.1 | 10.1 |
| **3h** | 2-Cl | 14 | 25 | 11.1 | 15.3 |
| **3i** | 4-Cl | 14 | 25 | 11.4 | 14.1 |
| **3j** | 2-Br | 14 | 25 | 12.1 | 16.5 |
| **3k** | 3-Br | 14 | 25 | 19.7 | 24.8 |
| **3l** | 4-Br | 14 | 25 | 20.3 | 26.5 |
| **3m** | 2-NO_2_ | 4 | 25 | 6.5 | 8.1 |
| **3n** | 3-NO_2_ | 4 | 25 | 7.4 | 10.6 |
| **3o** | 4-NO_2_ | 4 | 25 | 7.4 | 8.6 |
| **3p** | 2-CH_3_ | 4 | 25 | 12.1 | 14.6 |
| **3q** | 3-CH_3_ | 4 | 25 | 15.4 | 18.1 |
| **3r** | 4-CH_3_ | 4 | 25 | 14.9 | 18.4 |
| **3s** | 2-OCH_3_ | 4 | 25 | 11.3 | 14.1 |
| **3t** | 3-OCH_3_ | 4 | 25 | 13.9 | 17.1 |
| **3u** | 4-OCH_3_ | 4 | 25 | 14.1 | 17.3 |

**Table S8.** HPLC conditions and retention times for chiral analyses of **2**.

| **Compound** | | **MeOH**  **[%]** | **Temp.**  **[°C]** | **Retention time**  **[min]** | |
| --- | --- | --- | --- | --- | --- |
|  | R |  |  | **(*R*)*-*enantiomer** | **(*S*)*-*enantiomer** |
| **2a** | H | 14 | –8 | 73.8 | 57.2 |
| **2d** | 4-F | 14 | –8 | 82.8 | 63.9 |
| **2i** | 4-Cl | 14 | –8 | 252.1 | 214.7 |
| **2l** | 4-Br | 14 | –8 | – | – |
| **2r** | 4-CH_3_ | 14 | –8 | 168.4 | 143.7 |


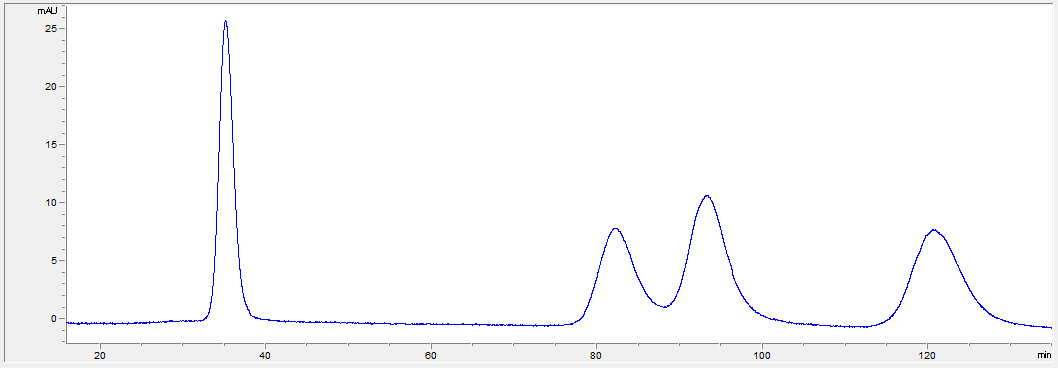


(*S*)-**3a**

(*S*)-**2a**

(*R*)-**2a**

(*R*)-**3a**


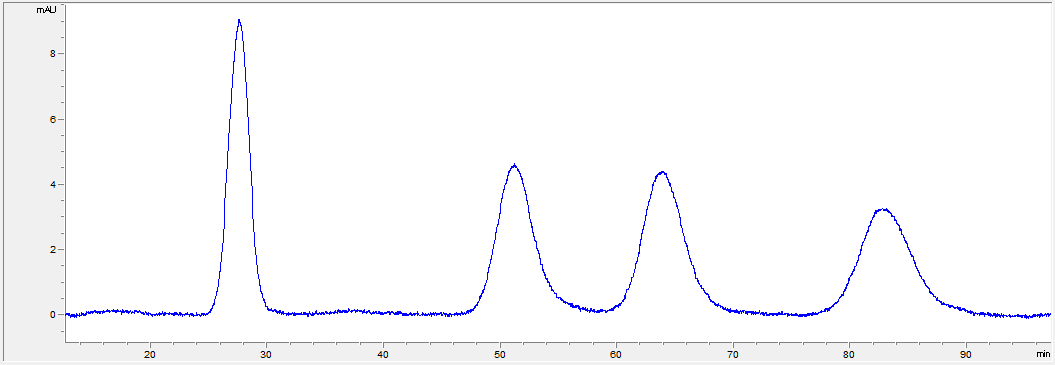


(*R*)-**2d**

(*S*)-**2d**

(*S*)-**3d**

(*R*)-**3d**


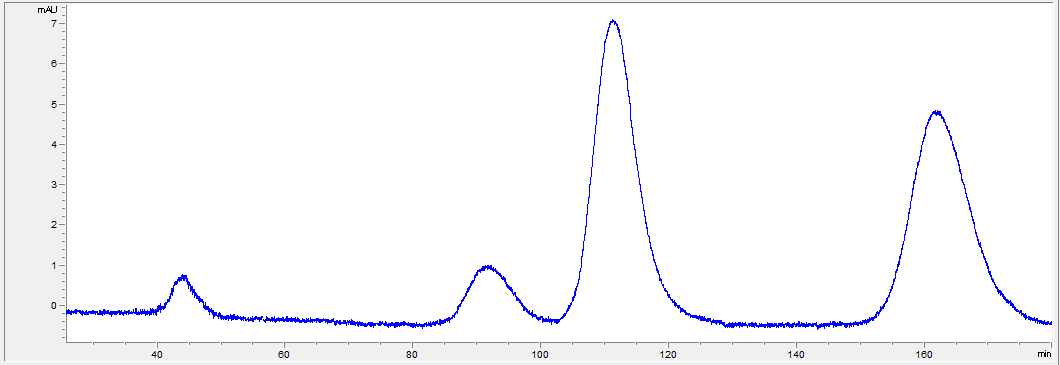


(*R*)-**2r**

(*S*)-**2r**

(*S*)-**3r**

(*R*)-**3r**

**Figure S2.** Representative HPLC chromatograms showing the separation of authentic standards of amino acid regioisomers and enantiomers.

# Preparative-scale production of β-amino acids with pyridylalanine substrates

Our work on the synthesis of substituted pyridylalanines revealed the propensity of AvPAL to produce the β-isomer. This effect was pronounced when we employed strongly electron-withdrawing halopyridylalanine substrates, for example with 2-chloro-4-pyridylacrylic acid and also, interestingly, with 2-methoxy-4-pyridylacrylic acid. Further investigation on a preparative scale revealed that prolonged incubation with AvPAL (>2 h) gave the β-regioisomers in a ratio of 41:59 (Figure S3a) with 2-chloro-4-pyridyl cinnamic acid and a ratio of 20:80 with the 2-methoxy-4-pyridyl cinnamic acid substrate (figure S3b).

(a)


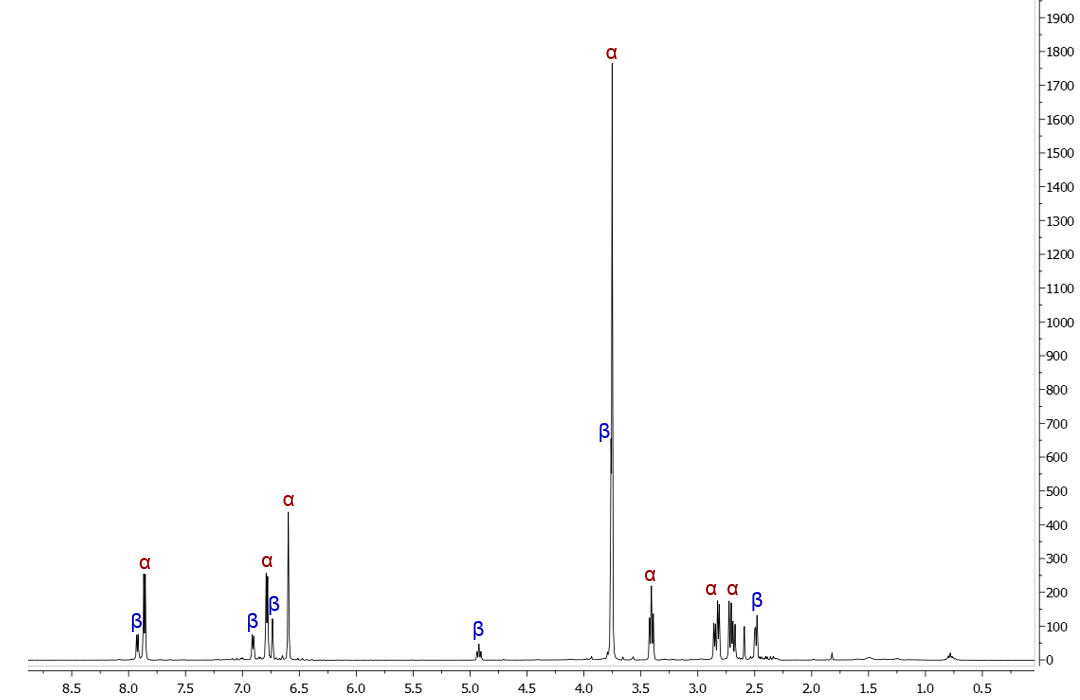

(b)


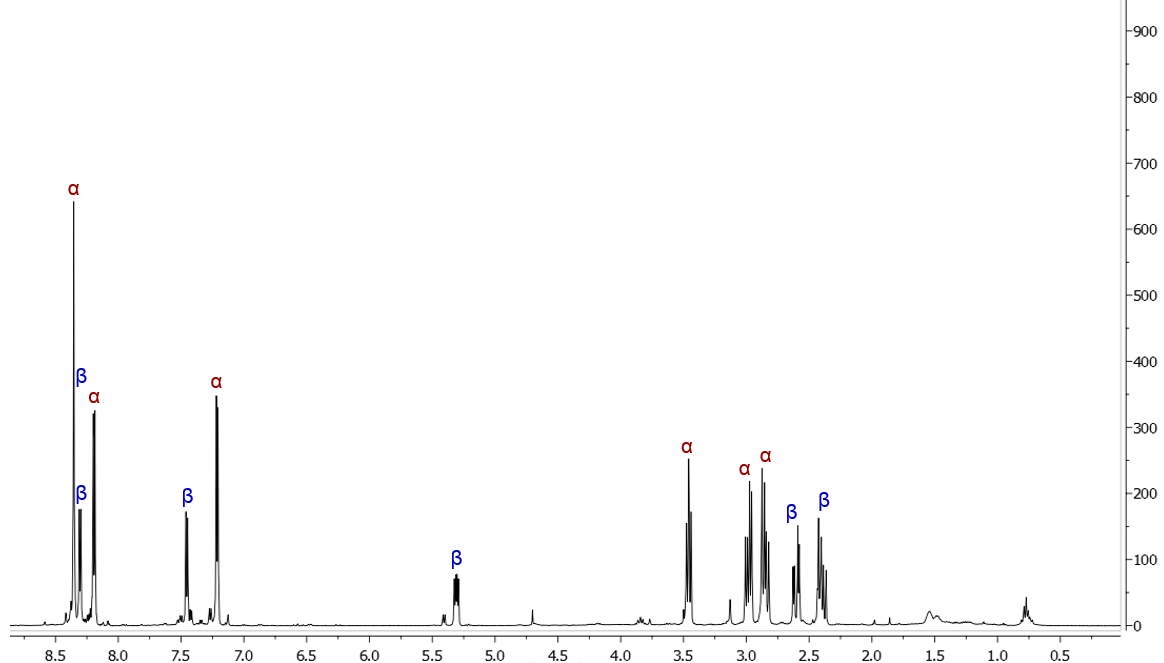

Figure S3. Preparative scale production of α- and β- regioisomers with 2-methoxy-4-pyridylacrylic acid (a) and 2-chloro-4-pyridylacrylic acid (b) after prolonged incubation with AvPAL at 37°C.

**References**

1. Lovelock SL, Lloyd RC, Turner NJ (2014) Phenylalanine Ammonia Lyase Catalyzed Synthesis of Amino Acids by an MIO-Cofactor Independent Pathway. Angew Chemie Int Ed 53:4652–4656. doi: 10.1002/ange.201311061
2. Parmeggiani F, Lovelock SL, Weise NJ, et al (2015) Synthesis of D- and L-Phenylalanine Derivatives by Phenylalanine Ammonia Lyases: A Multienzymatic Cascade Process. Angew Chem Int Ed Engl 54:4608–4611. doi: 10.1002/anie.201410670
3. Weise NJ, Parmeggiani F, Ahmed ST, Turner NJ (2015) The Bacterial Ammonia Lyase EncP: A Tunable Biocatalyst for the Synthesis of Unnatural Amino Acids. J Am Chem Soc 137:12977–12983. doi: 10.1021/jacs.5b07326
